# The effects of culturally adapted recruitment strategies for Black/ African Americans' engagement with Alzheimer’s Disease Research in New York City

**DOI:** 10.1002/alz.089524

**Published:** 2025-01-09

**Authors:** Laurel Y Browne, Omonigho Michael Bubu, Joanna Dominguez, Anamarie Sogade, Elizabeth Weisgerber, Anhiti Dharmapuri, Julian McBride, Oliver Cesar, Joshua L Gills, Fatoumata Barry

**Affiliations:** ^1^ NYU Grossman School of Medicine, Manhanttan, NY USA; ^2^ Center for Sleep and Brain Health, Department of Psychiatry, NYU Langone Health,, New York, NY USA; ^3^ NYU Grossman School of Medicine, New York, NY USA

## Abstract

**Background:**

In the United States, Black people represent 12% of the total US population and account for 19.3% of dementia cases. Social determinants of health (SDOH) and vascular comorbidities contribute to Black/African Americans having a higher risk of Alzheimer’s disease and related dementias (ADRD). More importantly, Black community underrepresentation in clinical research studies continues to be an issue. We aimed to employ culturally adapted recruitment methods to increase engagement of older Black/African Americans ages 55‐85 in ADRD research studies.

**Method:**

To mitigate the underrepresentation of Black/African Americans in ADRD studies, the Aging Research in Sleep Equity & Dementia Prevention Program (ARISE‐DP) at NYU Langone Health utilized multiple streams of recruitment with a focus on cultural inclusivity and humility. Recruitment methods included community outreach events, stakeholders, referrals, and recruitment companies (BuildClinical). In addition, a community liaison was utilized to form relationships, build trust, and increase engagement between the Black community in NYC & the ARISE‐DP Program. We compared previous recruitment methods, such as cold calls and mychart, to forming bonds and increasing literacy. The specific study’s recruitment goal for Black/African Americans was 125.

**Result:**

Recruitment efforts occurred from September 2023 to January 2024. As a result of the culturally adapted recruitment strategies, engagement with ARISE‐DP increased by 100%. Each strategy advanced the total number of people reached (N = 616): community outreach events (N = 297), stakeholders (N = 59), referrals (N = 34), and BuildClinical (N = 226). Individuals who expressed interest in participating in ARISE‐DP clinical trials were screened and, based on meeting eligibility criteria (age, cognition, and comfortability), were invited to be enrolled (N = 74) with a ratio of 44 Black women and 30 Black men recruited. BuildClinical (N = 22), community outreach events (N = 20), and stakeholders (N = 19) were the most effective recruitment strategies, followed by referrals (N = 12). As a result, we have met 59% of our recruitment goal in 3 months.

**Conclusion:**

Representation, education, culturally responsive methods, and including the community in recruitment increase trust and engagement in research studies. Recruitment strategies that acknowledge the historical barriers to Black/African American involvement in clinical studies and provide a holistic approach lead to an equitable, racially inclusive research environment.

